# The waxy mutation in sorghum and other cereal grains reshapes the gut microbiome by reducing levels of multiple beneficial species

**DOI:** 10.1080/19490976.2023.2178799

**Published:** 2023-02-21

**Authors:** Qinnan Yang, Mallory Van Haute, Nate Korth, Scott Sattler, Devin Rose, Anthony Juritsch, Jing Shao, Kristin Beede, Robert Schmaltz, Jeff Price, John Toy, Amanda E. Ramer-Tait, Andrew K. Benson

**Affiliations:** a Department of Food Science and Technology, University of Nebraska-Lincoln, Lincoln, NE, USA; b Nebraska Food for Health Center at the University of Nebraska, Lincoln, NE, USA; c Complex Biosystems Graduate Program, University of Nebraska-Lincoln, Lincoln, NE, USA; d Wheat, Sorghum and Forage Research Unit, USDA-Agricultural Research Service, Lincoln, NE, USA; e Department of Agronomy and Horticulture, University of Nebraska-Lincoln, Lincoln, NE, USA

**Keywords:** Waxy starch, resistant starch, sorghum, gut microbiome, butyrate, *Roseburia*, *Christensenellaceae*

## Abstract

Waxy starches from cereal grains contain >90% amylopectin due to naturally occurring mutations that block amylose biosynthesis. Waxy starches have unique organoleptic characteristics (e.g. sticky rice) as well as desirable physicochemical properties for food processing. Using isogenic pairs of wild type sorghum lines and their waxy derivatives, we studied the effects of waxy starches in the whole grain context on the human gut microbiome. *In vitro* fermentations with human stool microbiomes show that beneficial taxonomic and metabolic signatures driven by grain from wild type parental lines are lost in fermentations of grain from the waxy derivatives and the beneficial signatures can be restored by addition of resistant starch. These undesirable effects are conserved in fermentations of waxy maize, wheat, rice and millet. We also demonstrate that humanized gnotobiotic mice fed low fiber diets supplemented with 20% grain from isogenic pairs of waxy vs. wild type parental sorghum have significant differences in microbiome composition and show increased weight gain. We conclude that the benefits of waxy starches on food functionality can have unintended tradeoff effects on the gut microbiome and host physiology that could be particularly relevant in human populations consuming large amounts of waxy grains.

## Introduction

Starches are large polymers of glucose joined by glycosidic bonds and are important components of foods and food products worldwide. In the food industry, starch is introduced into a wide array of food products, but for most applications, the physicochemical properties of starches in their native form are unsuitable^1^. These undesirable behaviors of gelling, viscosity, and stability in native starches are typically overcome by modifying the starch through chemical or physical modification, crop breeding, or a combination of the two.^2^

Starches are made up of two major types of glucose polymers: amylose, an almost entirely linear (1,4)-α-D-glucan, and amylopectin, a highly branched α glucan containing both (1,4) and (16) linkages.^3^ Waxy starches were discovered in the 1900s in a unique, naturally occurring variant of maize that produced starches containing almost exclusively amylopectin.^4^ The absence of amylose in waxy starches produces desirable physicochemical properties (gelling, viscosity, and stability), and waxy starches are widely used by the food industry as thickeners, gelling agents, and stabilizers.^5^ Since the discovery of the waxy phenotype in maize, naturally occurring waxy variants have also been identified in many other grain crops, including sorghum, rice, proso millet, and wheat. In the cases that have been studied, causal variants of the waxy phenotypes in cereal grains are due to loss of function mutations in the gene encoding granule bound starch synthase (*GBSS*), the enzyme responsible for amylose synthesis in starch granules.^6–12^

In addition to the effects of waxy mutations on physicochemical properties of starch, the reduced amylose in waxy derivatives also has significant effects on starch digestibility. The branched structure of amylopectin increases sites for enzymatic digestion and resists the effects of retrogradation when thermally processed.^13^ In humans, the high digestibility amylopectin leads to nearly complete digestion of waxy starches by host amylases in the upper gastrointestinal (GI) tract.^14,15^ Unfortunately, this increased digestibility is also associated with a higher glycemic index rating for waxy starches compared to nonwaxy wild type starches.^16,17^ The condensed straight chain structure of amylose has fewer sites for degradation by host enzymes, and consequently, a sizable portion of ingested amylose survives transit through the small intestine and enters the colon where it serves as a substrate for fermentation by amylolytic organisms in the colonic microbiota.^18,19^

Resistant starches (RS), which are recalcitrant to hydrolysis and enzymatic digestion, have been shown to provide health benefits to humans, including improved glucose tolerance and cholesterol levels as well as reduced inflammatory markers and toxic biomarkers for chronic kidney disease.^20–23^ Although amylose concentration influences the digestion resistance of RS, other factors such as source, granule composition, and thermal versus chemical modification also contribute and are used to classify RS into different categories.^13^ RS mediates metabolic improvements through microbiome dependent and independent pathways.^24–26^ The microbiome dependent pathways appear to be mediated through RS-stimulated growth of beneficial amylolytic bacteria that metabolize RS to short-chain fatty acids (SCFA) such as butyrate.^27,28^ The effects of microbially produced butyrate are pleiotropic and afford the host multiple benefits such as providing energy to colonocytes, enhancing anti-inflammatory functions, and protecting against allergic responses.^25,26^ Thus, in addition to promoting high glycemic responses, the absence of amylose in waxy starches presumably leads to lower levels of RS, consequently reducing RS-mediated benefits mediated by the colonic microbiota.

Although resistant starch has beneficial effects on both the gut microbiome and characteristics related to gut health, most of the relationships between RS and human health have been studied in the context of pure RS. We therefore know very little about the relationships of waxy starches in their native, whole-grain context with the microbiome and host health. This gap is particularly relevant in Asia and Africa, where rice and sorghum are consumed daily as whole grain staples. While studies of locally grown and consumed rice and sorghum are limited, recent work on locally grown and consumed rice cultivars in selected areas of Asia has shown that nearly half of the cultivars studied have low amylose content and would be considered waxy.^29,30^ Therefore, understanding how waxy phenotypes, in the whole grain context, may influence the microbiome and host physiology is of primary interest to domestic and global health.

To address the knowledge gaps related to effects of waxy grains on the gut microbiome in a whole-grain context, we used near-isogenic lines of wild type and waxy derivatives of sorghum to study the effects of whole-grain starch composition on the human gut microbiome. Using a combination of *in* *vitro* microbiome fermentations and mouse feeding studies, we detected significant differences in the effects of waxy derivatives versus wild type parental lines on the overall composition of the microbiome and the abundances of several microbial taxa, many of which corresponded to significant decreases in abundances of beneficial microbes in treatments with waxy lines. In addition to the strong signatures of effects on the microbiome, our studies with human microbiota-associated mice also demonstrated dramatic effects of waxy starch on host weight gain, with significant increases in weight gain observed among animals fed diets with waxy versus wild type parental sorghum. Collectively, our work highlights the need to understand how traits such as waxy, which have major effects on grain composition, can have strong trade-off effects on the gut microbiome and host health characteristics.

## Results

### *In vitro* fermentation of near-isogenic lines of wild type and waxy sorghum grain with human stool microbiomes yielded distinct effects on microbiome diversity

In the first set of experiments, we examined the outcomes of *in* *vitro* microbiome fermentation reactions across a set of near isogenic lines (NILs) of sorghum derived from six different elite genetic backgrounds. Amylose and resistant starch content (total starch content after *in* *vitro* digestion and dialysis) from each pair of NILs was measured (Table 1). Across the six pairs of lines used for our studies, the wild type lines yielded 7-13 times more amylose and 3–5 times more resistant starch (0.21–0.39%) than their waxy derivative (0.07–0.1%). Indeed, the waxy mutation significantly increased the starch digestibility, leaving less resistant starch for microbial fermentation. Grain from each of these six pairs of wild type lines and isogenic waxy derivatives was subsequently used in individual *in* *vitro* microbiome fermentation reactions with human stool microbiomes from 12 donors (three females and nine males) with distinct baseline microbiome compositional features (Figure S1).

**Table 1.** Information for the grain lines used in this study.

Commodity	Line	Starch type	Amylose content % (w/w)	Resistant starch content % (w/w)	Source
Sorghum	N619	Waxy	1.02	0.10	Provided by Scott Sattler ^8^
	N620	Wild type	9.80	0.21	
	N621	Waxy	0.83	0.07	
	N622	Wild type	11.30	0.35	
	N625	Waxy	0.89	0.08	
	N626	Wild type	10.55	0.25	
	N639	Waxy	1.04	0.07	
	N640	Wild type	12.58	0.27	
	RN642	Waxy	0.89	0.07	Provided by Scott Sattler^31^
	Tx430	Wild type	11.73	0.34	
	BN461	Waxy	0.79	0.08	
	Wheatland	Wild type	13.27	0.40	
Rice	Japanese sweet rice	Waxy	0.72	0.05	Local grocery store
	Jasmine rice	Wild type	7.43	0.11	
Wheat	Waxy wheat	Waxy	0.78	0.09	From UNL wheat quality lab^11^
	Non-waxy wheat	Wild type	11.99	0.81	
Millet	Plateau	Waxy	0.61	0.08	Provided by Ismail Dweikat^10^
	Hunksman	Wild type	12.13	0.54	
Maize	W64A waxy	Waxy	0.81	0.07	Ordered from Genetic Resource Collections^32^
	W64A WT	Wild type	9.74	0.47	
	K55 waxy	Waxy	0.69	0.06	
	K55 WT	Wild type	11.43	0.35	

**Figure 1.**
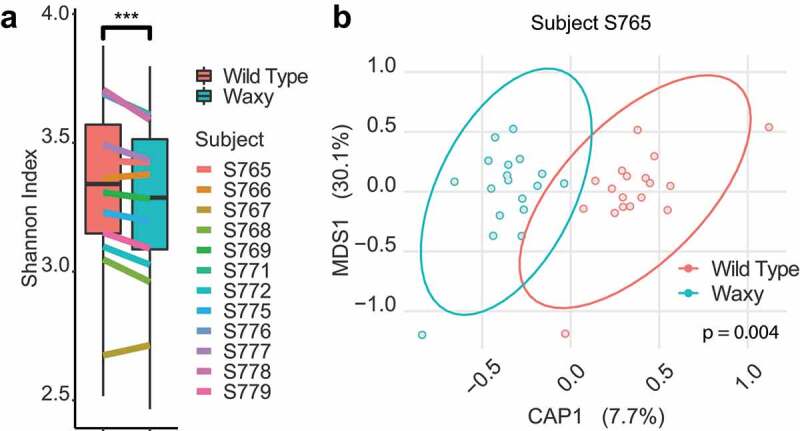
Effects of waxy sorghum on fecal fermentation bacterial diversity. (a) Box plots of Shannon index for the microbiomes from each subject after fermentation between wild type and waxy sorghum. Lines with different colors represent the average Shannon index of different subjects; paired Wilcoxon test, *p* < .001: ***. (b) Canonical analysis of principal coordinates (CAP) plot based on Bray–Curtis distance, showing the overall microbiome composition difference between waxy and wild type sorghum in subject S765 (*p*-value was calculated using PERMANOVA).

Compositional features of the microbiomes from multiple subjects showed highly significant treatment effects in fermentations of waxy compared to wild type parental lines. These treatment effects (waxy versus wild type) manifested as differences in ecological metrics (α- and β-diversity) of the microbiomes as well as significant differences in the relative abundances of individual and groups of taxa. With respect to ecological metrics, the Shannon index (α-diversity) was significantly lower in fermentations with waxy sorghum when compared to wild type lines (Figure 1a). PERMANOVA of Bray–Curtis distances also showed significant differences in β-diversity (*p* < .001). Subsequent analysis of β-diversity in samples from each individual microbiome by canonical analysis of principal coordinates (CAP) based on Bray–Curtis distance illustrated the strength of associations between β-diversity of the microbiomes with wild type or waxy grain lines (Figures 1b and S2), and PERMANOVA of Bray Curtis distance further highlighted the statistical differences in overall microbiome composition in the microbiomes from all 12 donors (*p* < .05, Figure S2). When compared to fermentations of the wild type parental lines, our results collectively show that the waxy lines had major effects on the overall α- and β-diversity of the microbiomes, and that microbiomes from each subject were able to differentiate substrates from wild type versus waxy sorghum regardless of the sorghum genetic background in which the waxy mutations were introduced.

### Taxonomic features of the human gut microbiome from *in* *vitro* fermentation of wild type and waxy sorghum revealed shared and individualized patterns of responsiveness among different human donors

**Figure 2.**
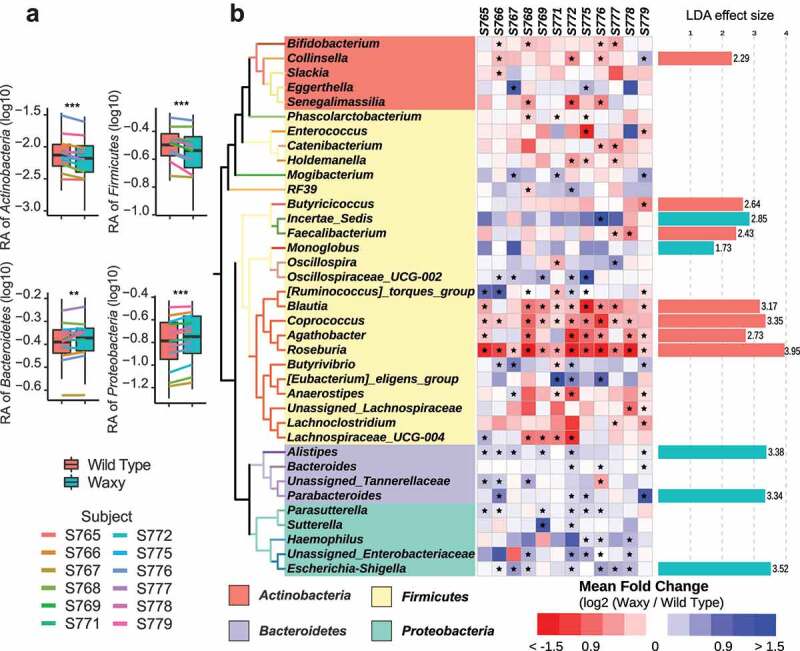
Shifts in the abundance of bacterial taxa between waxy and wild type sorghum. (a) Box plots of the relative abundances of bacterial phyla and one family that showed the most significant difference between waxy and wild type sorghum; lines with different colors representing the average abundance of specific taxa in different subjects (rANOVA followed by FDR correction, *p* < .05: *; *p* < .01: **; *p* < .001: ***). (b) Heatmap of the mean log2-transformed fold change of genera that showed significant effects of wild type sorghum relative to waxy sorghum in one or more subjects. Statistical significance of changes between wild type and waxy sorghum were determined by applying two-way rANOVA (with FDR correction); q < 0.05 considered significant and denoted by asterisk. LEfSe effect size showing genera that were enriched in either wild type or waxy sorghum. The LDA effect size is colored orange (greater in wildtype) or green (greater in waxy).

Given the highly significant effects of the waxy versus wild type parental grain phenotypes on both α and β-diversity metrics of microbial communities, we next identified specific microbial taxa associated with the treatment effects (waxy versus wild type) from each donor microbiome. Statistical significance was tested at multiple taxonomic levels. At the phylum level, significantly higher abundances of *Proteobacteria* and *Bacteroidetes* were found in fermentation of waxy lines, whereas significantly higher abundances of *Firmicutes* and *Actinobacteria* were found in fermentations from wild type lines (paired Wilcoxon test followed by FDR correction, Figure 2a). The trends stayed much the same at increasing levels of taxonomic resolution. At the genus level, we detected three or more genera that accounted for many of the differences at the phylum level (paired Wilcoxon test followed by FDR correction, Figure 2b). For example, within the phylum *Bacteroidetes*, the genera *Allistipes, Bacteriodes, Parabacteriodes*, and an unclassified taxon in *Tannerellaceae* each showed the same trend (greater abundances in fermentations of waxy lines) with statistical significance in at least three of the subject microbiomes. Similarly, *Sutterella, Escherichia-Shigella*, and an unclassified taxon from *Enterobacteriaceae* were each present at significantly higher abundances in fermentations from waxy lines from microbiomes of five or more donors. Accounting for most of the significant increase in *Firmicutes* from fermentations of wild type parental lines were members of the family *Lachnospiraceae*, and genera from this family also showed the most consistent behavior across donor microbiomes. For example, *Roseburia* was significantly higher in fermentations of wild type sorghum across all 12 donor microbiomes while *Blautia* and *Coprococcus* were significantly higher in nine and ten microbiomes (Paired Wilcoxon test followed by FDR correction, Figure 2b). Independent analysis of the data by linear discriminant analysis effect size analysis (LEfSe) also identified similar bacterial taxa with the greatest contribution to treatment effects (waxy versus wild type; Figure 2b). LEfSe identified *Escherichia-Shigella* and *Alistipes* as the major taxonomic groups driving fermentations of waxy lines whereas the genera driving fermentations of wild type sorghum lines corresponded to members of the *Lachnospiraceae*, namely, *Roseburia, Coprococcus*, and *Blautia*.

### Individual species of *Roseburia* are highly responsive to amylose content across different human donors

**Figure 3.**
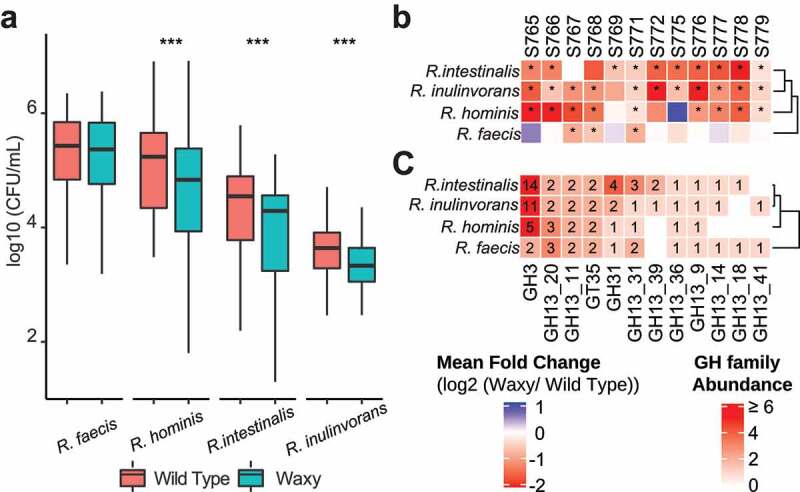
Characterization of *Roseburia* species between waxy and wild type sorghum fermentations. (a) Box plots of the absolute abundance of different *Roseburia* species in waxy and wild type sorghum after fermentation (rANOVA followed by FDR correction, *p* < .05: *; *p* < .01: **; *p* < .001: ***). (b) Heatmap of the mean log2-transformed fold change of different *Roseburia* species in wild type sorghum relative to waxy sorghum in the microbiome of each subject. Statistical significance of changes between wild type and waxy sorghum were determined using two-way rANOVA (with FDR correction); *p* < .05 considered significant and denoted with asterisk. (c) Heatmap of starch degradation-associated GH family abundance in different *Roseburia* species.

The most consistent microbiome-wide treatment effect (waxy versus wild type) across hosts corresponded to increased abundances of the amylolytic genus *Roseburia* in fermentations of grain from wild type parental versus waxy lines. Using species-specific qPCR reactions, we confirmed the observations from the 16S rRNA sequencing data and found that this behavior was shared by three of the major *Roseburia* species from human microbiomes (*R. intestinalis, R. hominis*, and *R. inulinivorans*) as each of these species were significantly enriched in fermentations from wild type parental sorghum lines across microbiomes from ten or more subjects compared to fermentations from near-isogenic waxy derivative lines (Figure 3a and b). *R. faecis* was much less enriched, showing significant differences in only three out of twelve subjects (Figure 3a and b).

Comparisons of CAZyme glycohydrolase families (GH) found in the genomes of representative strains of four human *Roseburia* species offers an explanation as to why *R. intestinalis, R. hominis*, and *R. inulinovorans* were more responsive to differences in amylose content compared to *R. faecis*. Genomes of *R. intestinalis, R. hominis*, and *R. inulinivorans* had 5–14 different genes encoding GH3-family enzymes associated with starch degradation whereas *R. faecis* carried only two genes encoding GH3 enzymes (Figure 3c). Thus, enrichment of the GH3 enzyme family in *R. intestinalis, R. hominis*, and *R. inulinivorans* may provide a selective advantage for growth on starch-rich substrates and may explain why these three species were more responsive to treatment effects (waxy versus wild type) across individual microbiomes.

### Waxy sorghum leads to reduced butyrate production in *in* *vitro* fermentations with microbiomes from multiple human donors

**Figure 4.**
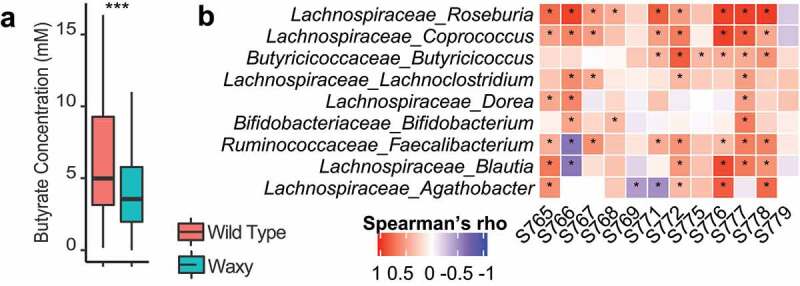
Waxy sorghum altered SCFA production. (a) Box plot of the concentration of butyrate in waxy and wild type sorghum (rANOVA followed by FDR correction, *p* < .001: ***). (b) Heatmap shows the association between butyrate production and relative abundance of specific bacterial genera in each subject using Spearman’s correlations; statistical significance of the association was corrected with FDR correction; *p* < .05 considered significant and denoted by asterisk.

The significant decreases in the abundances of amylolytic, butyrate-producing members of the *Lachnospiraceae (*e.g., *Roseburia)* in fermentations of waxy lines across multiple microbiomes would also be expected to be accompanied by decreased production of butyrate, the major end-product of starch fermentation by these organisms. Measurement of the major SCFAs by gas chromatography of supernatants from the fermentations (Figure 4a) confirmed this hypothesis, with significantly lower concentrations of butyrate in fermentations from waxy lines compared to wild type (average of 24% decrease, *p* < .001, rANOVA followed by FDR correction). Concentrations of the other major SCFA (acetate, propionate, isobutyrate and isovalerate) were not significantly affected by treatment (waxy versus wild type lines).

Correlation analysis (Spearman’s correlation) further supports substantial roles for members of the *Lachnospiraceae* in butyrate production as the relative abundances of *Roseburia* (9 out of 12 microbiomes) and *Coprococcus* (8 out of 12 microbiomes) had some of the strongest correlations with butyrate production (Figure 4b, Spearman’s correlation with FDR correction). Notably, *Butyricicoccus, Blautia*, and *Faecalibacterium* also showed significant correlations with butyrate production in at least five different microbiomes (Figure 4b). Thus, while butyrate production in these fermentations is polymicrobial, members of the *Lachnospiraceae* family, particularly *Roseburia* and *Coprococcus*, seem to have the most significant microbiome-wide contributions to butyrate production and these taxa are known to possess pathways for fermentation of glucose to butyrate,^33^ thus suggesting that they are efficient at utilizing amylose present in the wild type parental sorghum lines.

### Resistant starch extracted from wild type sorghum lines restore changes in microbiome phenotype with waxy sorghum

**Figure 5.**
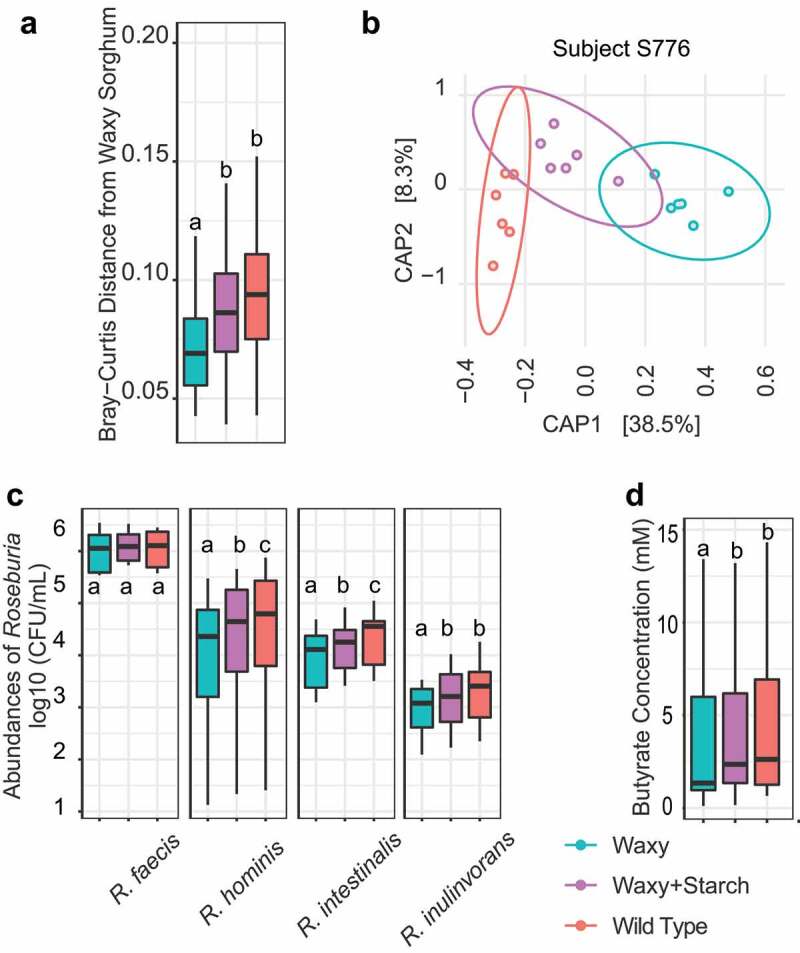
Wild type starch restored microbiome features in waxy sorghum, and wild type starch shows dose-dependent effects on bacterial composition and function. (a) Box plot of Bray–Curtis distances between each group to waxy sorghum group; data were analyzed via a Kruskal–Wallis test followed by post hoc pairwise multiple comparisons using Dunn’s test with FDR corrections. (b) Canonical analysis of principal coordinates (CAP) plot based on Bray–Curtis distance showing the overall microbiome composition difference across three substrate groups in subject S766 (see Supplemental Figure 3 for additional analyses on Bray–Curtis distance of samples from other subjects). (c) Box plots of the absolute abundance of different *Roseburia* species as measured by qPCR in three treatments; data were analyzed via rANOVA followed by FDR correction. (d) Box plots of concentration of butyrate in three treatments; data were analyzed via rANOVA followed by FDR correction. Significant differences are denoted by different letters (*p* < .05). (e) Spearman’s correlations between starch doses and either the concentrations of butyrate, the abundances of selected genera, or *Roseburia* species (FDR correction; *p* < .05: *).

Mutations affecting the synthesis of major seed components (i.e., starches in the endosperm) can also have pleiotropic effects on other major components of the seed and further impact microbiome (i.e., protein content).^34,35^ We therefore used “molecular complementation” experiments to confirm that the differential microbiome effects observed in fermentation of whole grain from wild type and waxy lines were due to amylose content alone and not an unknown pleiotropic effect of the GBSS mutations on other seed components. Molecular complementation was achieved by introducing digestion-resistant starch purified from wild type sorghum lines into fermentations with grain from amylose-deficient waxy lines and examining effects on microbiome phenotypes. Microbiome data from the fermentations were analyzed by comparing Bray–Curtis distance of microbiomes from (i) fermentation of waxy sorghum lines alone, (ii) fermentations of waxy lines supplemented with amylose-enriched (digestion-resistant) starch from wild type lines, and (iii) fermentations of wild type lines alone. The addition of the digestion-resistant starch from wild type lines indeed caused significant shifts in β-diversity in the microbiomes across multiple human subjects, with microbiomes from most subjects responding to complementation with profiles that were intermediate to profiles from fermentation of wild type parental or waxy lines (Figures 5 and S3) but demonstrating statistically significant responses to complementation (Kruskal–Wallis test followed by post hoc pairwise multiple comparisons using Dunn’s test; Figure 5a).

We further investigated molecular complementation at higher taxonomic resolution by qPCR quantification of *Roseburia* species in the fermentation reactions (Figure 5c). These reactions showed significant increases in the abundance of *R. intestinalis, R. hominis*, and *R. inulinivorans* but not *R. faecis* in the fermentations from waxy lines complemented with resistant starch from wild type lines compared to fermentations from waxy sorghum lines (Kruskal–Wallis test followed by post hoc pairwise multiple comparisons using Dunn’s test, Figure 5c). Molecular complementation of resistant starch from digestion into fermentations with waxy sorghum lines also produced an expected stimulation in butyrate production, similar to the levels observed during fermentations with wild type lines (rANOVA followed by FDR correction, Figure 5d). Thus, introduction of amylose-enriched, digestion-resistant starch from wild type parental lines into fermentation reactions of waxy lines promotes restoration of microbiome profiles in fermentations from waxy lines to profiles that are observed from fermentations of wild type parental sorghum lines, including stimulation of some of the most responsive amylolytic taxa (*Roseburia*) and concomitant changes in butyrate production. Consequently, the significant microbiome phenotypes caused by the waxy mutation in our *in* *vitro* fermentation reactions appear to be primarily dependent on the effects of waxy mutations on amylose content of the grain.

### Waxy starch has similar effect on microbiome in many small grain commodities

The waxy starch trait has been developed in many small grain plant species due to its unique physicochemical properties. As with our sorghum lines, waxy lines of these other small grain species have a lower concentration of digestion-resistant starch compared to corresponding wild type lines after digestion (Table 1). To determine if the waxy trait in a whole-grain context from other species of small grains shows similar effects on the microbiome as we observed in sorghum, we compared *in* *vitro* fecal fermentations on grain derived from waxy and wild type lines of sorghum, maize, millet, rice, and wheat using the same 12 donor microbiomes for all grains tested. While the sorghum and maize grain for this experiment were derived from NILs of wild type parental and waxy derivatives, grain from wheat, rice, and millet were not derived from isogenic pairs.

**Figure 6.**
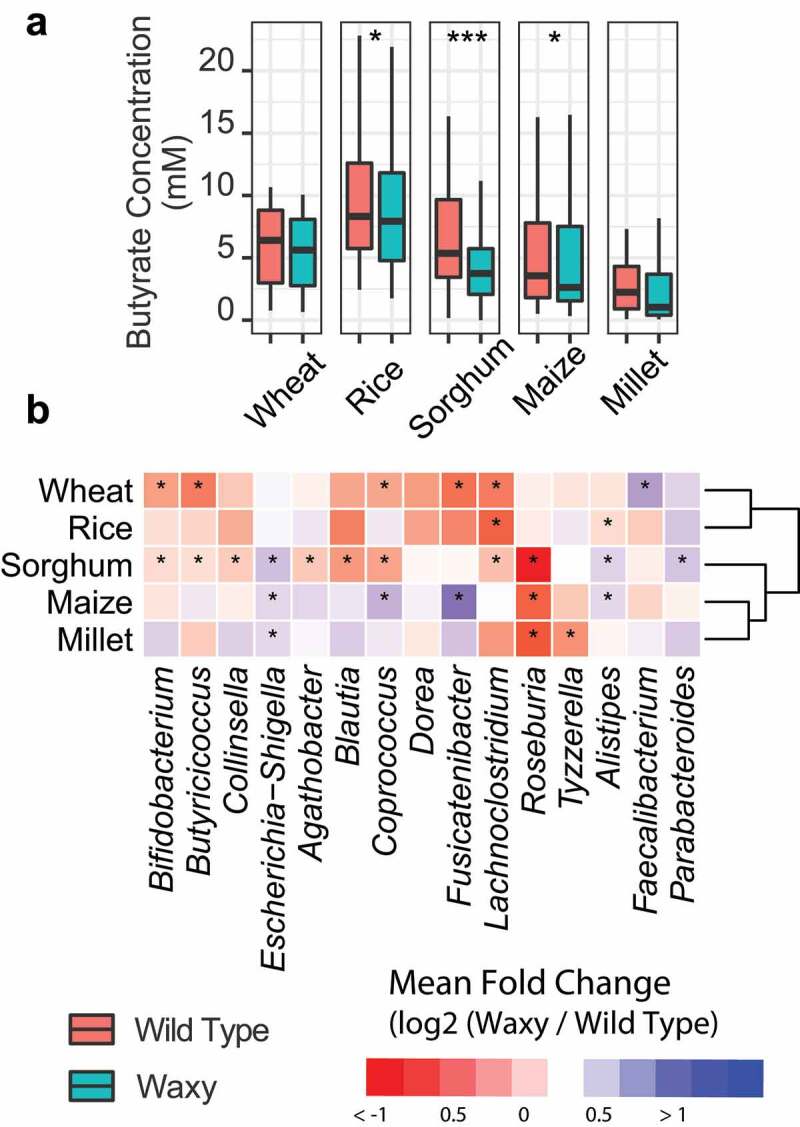
Effect of the waxy trait in different species of small grains. (a) Box plots of butyrate concentration in different waxy and wild type commodities after *in* *vitro* fermentation; data were analyzed via paired Wilcoxon test, *p* < .05: *; p < .01: **; *p* < .001: ***. (b) Heatmap of the mean log2-transformed fold change of bacterial genera in wild type commodities relative to corresponding waxy commodities; data were analyzed via two-way rANOVA with FDR correction; *p* < .05: *.

Combined data from all 12 human microbiomes showed significant reduction of butyrate production in fermentations of waxy grain from rice, sorghum, and maize with the most significant reduction occurring between waxy and wild type lines of sorghum (paired Wilcoxon test, Figure 6a). Microbiome analysis revealed significant abundance differences in many genera (Figure 6b) and, like the butyrate production data, microbiome responsiveness to waxy and wild type lines of sorghum showed the most significant taxonomic responses based on the number of taxa showing statistically significant responses. A small number of microbial taxa showed shared responses to wild type versus waxy fermentations across multiple crop species (e.g., waxy grain from sorghum, maize, and millet all yielded significant reductions in abundances of *Roseburia* and elevated abundances of *Escherichia* based on two-way rANOVA with FDR correction). However, the taxonomic responses were largely unique to each of the crop species (Figure 6b). The unique effects of waxy wheat and rice may be due in part to the lines not being isogenic (e.g., contribution from variation at other genetic loci) and/or differences in the penetrance of the waxy mutations or differences in the physicochemical characteristics of amylose from those species (e.g., different degrees of polymerization). The important finding, however, is that human microbiomes appear to display significantly different fermentation patterns of grain from waxy versus wild type lines from each of the crop species and further experimentation is clearly warranted to understand relationships between waxy phenotypes in these crop species, physicochemical characteristics of the starches, and their impacts on fermentation by gut microbes.

### The waxy phenotype in whole grain sorghum alters the gut microbiome and affects weight gain in human microbiome-associated mice

**Figure 7.**
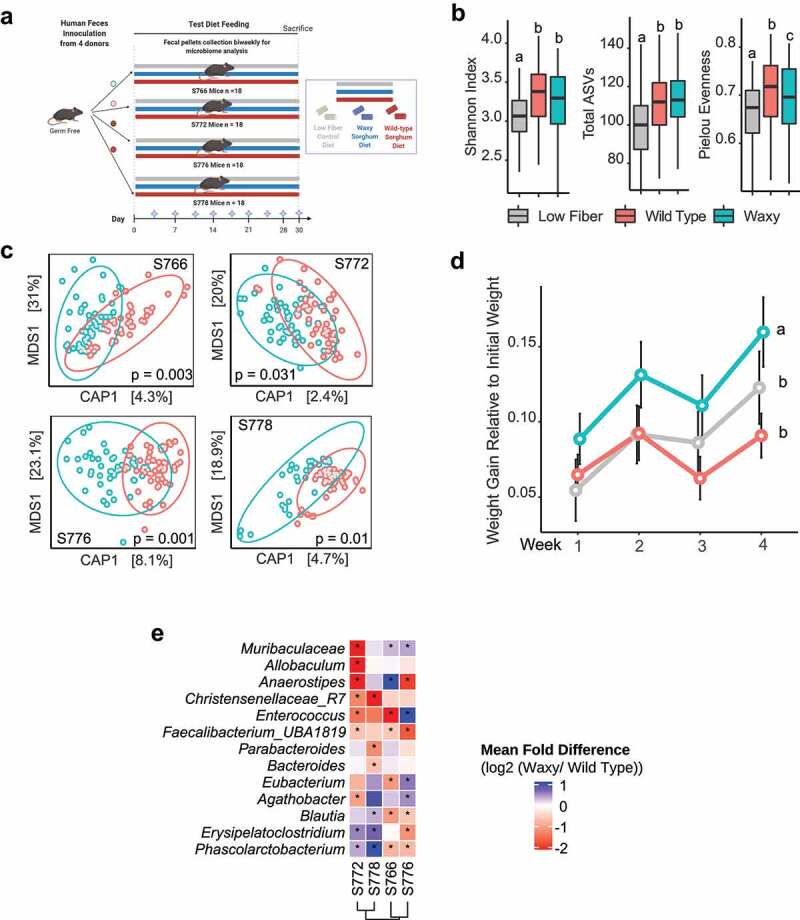
Effects of waxy sorghum on human microbiome-associated (HMA) mice. (a) Experimental design for mouse feeding study. Circles above the arrows represent each of the four microbiomes. The gray diamonds on the X-axis indicate microbiome sampling points. (b) Box plots of the Shannon index, total ASVs, and Pielou Evenness metrics for fecal and cecal microbiomes of mice fed each of three diets; data were analyzed via rANOVA followed by FDR correction. Groups showing statistical significance (P<0.5) are indicated by letter (c) Canonical analysis of principal coordinates (CAP) plot based on Bray–Curtis distance showing the overall microbiome composition differences between mice fed either waxy or wild type sorghum and harboring donor microbiome S776 (p and R^2^ values were calculated using PERMANOVA, see Supplemental Figure 3 for additional analyses on Bray–Curtis distance of samples from other subjects). (d) Body weight relative to initial weight over time; data are presented as mean ± SEM; data were analyzed via rANOVA followed by FDR correction. Significant differences are denoted by different letters (*p* < .05). Heatmap of the log2-transformed fold change of bacterial genera in the four HMA mouse lines consuming wild type sorghum diets vs waxy sorghum diets; data were analyzed via two-way rANOVA with FDR correction; *p* <0.05

Although *in* *vitro* fermentation is an excellent model for estimating the capacity of different substrates to influence the microbiome, assessing the extent to which such substrates induce similar microbiome changes in an animal model represents a critical pre-clinical step toward translation to humans. Colonizing germ-free mice with human fecal microbiomes to generate human-microbiome-associated (HMA) mice enables study of the functional consequences of dietary components on human-adapted gut microbes within a model animal system.^36,37^ To that end, we used HMA mice to determine if feeding diets supplemented with 20% whole grain flour from either isogenic wild type or waxy sorghum lines could drive significant microbiome changes in vivo. Four unique HMA mouse lines were created by colonizing germ-free C57BL/6 mice with one of four human microbiomes (S766, S772, S776, and S778) demonstrating the most significant differential responses to the sorghum substrates during *in* *vitro* fermentation studies (Figure 7a). After introduction of the microbiomes, the HMA lines were divided into three treatment groups of six animals per treatment per HMA line. Among the treatments within an HMA line, one was fed a low-fiber diet while the others were fed diets supplemented with 20% sorghum from either the wild type line or the isogenic waxy derivative.

Characterization of fecal (eight time points) and cecal (terminal time point) bacterial communities of HMA mice by 16S rRNA gene sequencing over time revealed that both wild type and waxy sorghum diets increased the α-diversity (Shannon index, total ASVs, and Pielou’s evenness index) of the bacterial community compared to a low fiber control diet. There were no significant differences in the Shannon index or number of ASVs in the microbiomes of mice fed waxy or wild type sorghum diets, but a lower Pielou’s evenness index was observed in mice fed waxy sorghum (Kruskal–Wallis test followed by post hoc pairwise multiple comparisons using Dunn’s test, Figure 7b). In contrast, constrained ordination analysis of Bray-Curtis distances showed significant differences (*p* < .05, PERMANOVA) in the β-diversity of HMA mouse microbiomes from all four human donors when fed a waxy sorghum diet compared to a wild type sorghum diet (Figure 7c). Thus, even with sorghum representing only 20% of the diet, the differences in amylose content drove significant treatment effects (wild type versus waxy sorghum) in the microbiomes of all four HMA mouse lines.

Consistent with the changes in β-diversity, significant differences in the relative abundances of bacterial genera were also detected in each HMA mouse line fed diets containing waxy versus wild type sorghum (paired Wilcoxon test followed by FDR correction, Figure 7e). The patterns of taxa showing statistically significant treatment effects (waxy versus parental) were unique to each individual microbiome, a result commonly observed in experiments using HMA mice that harbor different donor microbiomes.^38–40^ In HMA mice carrying the stool microbiome of subject S772, taxa that were significantly less abundant in animals fed the waxy sorghum diet included members of the *Bacteriodales* (*Muribaculaceae), Erysipelotrichiaceae (Allobaculum), Lachnospiraceae (Agathobacter* and *Anaerostipes), Ruminococcaceae* (*Faecalibacterium), Clostridia* (*Christensensellaceae*) and *Enterococcaceae* (*Enterococcus*) whereas abundances of *Erysipeloatoclostridium* (*Erysipelotrichaceae)* and *Phascolarctobacterium* (*Acidaminococcaceae*) were higher in mice fed waxy sorghum. Microbiomes from HMA mice harboring stool from subject S778 shared some overlapping taxonomic responses with those carrying the S772 microbiota when receiving waxy sorghum compared to wild type sorghum, including decreased abundances of *Christensenellaceae* and increased abundances of *Erysipeloatoclostridium* and *Phascolarctobacterium*. Another intriguing aspect of the microbiome-dependent responses was the observation that treatment effects on several of the significant taxa were not necessarily in the same direction for each microbiome, implying that the microbiome context is a major determinant of how individual microbial taxa may respond. However, we did note that three taxa, *Faecalibacterium, Christensenellaceae*, and *Enteroco*c*cus*, exhibited significant diet-driven responses in the same direction (e.g., decreased in waxy sorghum diet) across HMA mouse lines from two or more donor microbiomes.

Although dietary treatments (wild type versus waxy) had significant effects on compositional features of the microbiomes in HMA mouse lines, including members of the *Lachnospiraceae*, a notable difference between responses of the microbiomes in the *in* *vitro* fermentations compared to the HMA feeding experiment was the absence of treatment effects on *Roseburia* in the HMA mice. Given the strong responsiveness of this organism to wild type versus waxy grain in the *in* *vitro* experiments and the consistency of this response across multiple human microbiomes (Figure 2), absence of significant treatment effects in the HMA mice was unexpected. These disparate results are explained, however, by inefficient colonization of *Roseburia* species, as qPCR assays for *Roseburia* species (Figure S4) showed that populations of *Roseburia* species declined rapidly after microbiome introduction in all four HMA lines. Indeed, by day 7, populations of all four *Roseburia* species declined to levels near the threshold for detection, suggesting that *Roseburia* did not efficiently engraft and persist in ex-germ-free mice. Further study is needed to determine if poor colonization of the mouse host is a general feature of *Roseburia*.

In addition to the microbiome phenotypes, we also tested for treatment effects (wild type versus waxy) on feed intake and weight gain throughout the study. Remarkably, we found that animals from all four HMA mouse lines fed diets supplemented with waxy sorghum gained significantly more weight compared to their counterparts receiving either the wild type sorghum or low fiber control diets (rANOVA followed by FDR correction, Figure 7d). These weight gain phenotypes were observed in the absence of any significant treatment effects on feed intake (Figure S5), and are thus driven by compositional differences (e.g., amylose content) between the diets, most likely by more efficient digestion of the higher levels of amylopectin in feed derived from waxy lines.

To test for potential diet-driven effects on host immune responses, we evaluated cytokine and chemokine levels for all HMA mice in each dietary treatment. Quantification of 32 cytokines and chemokines in the sera of mice after four weeks of feeding test diets revealed significant effects of diet on levels of IL-5, KC (mouse IL-8), and MCP-1 (rANOVA followed by FDR correction; Figure S6 and Table S1). Mice receiving a wild type parental sorghum flour diet had significantly lower levels of IL-5, KC, and MCP-1 compared to mice fed a low fiber diet. In comparison, feeding a waxy sorghum diet induced intermediate levels of IL-5, KC, and MCP-1 that were not significantly different compared to those observed when feeding either a wild type parental sorghum or low fiber diet (rANOVA followed by FDR correction). Together, these data demonstrate that even within a relatively short feeding period and in the absence of a disease state, there is evidence for low-levels of diet-induced immune modulation in HMA mice.

## Discussion

Although waxy grains and starches are widely consumed,^5,41–43^ little is known about how these starches affect the gut microbiome or other characteristics related to human health. To begin filling this knowledge gap, we used a combination of *in* *vitro* fermentation and preclinical animal model to assess effects of whole grain from parental lines versus their cognate waxy derivatives on compositional and metabolic features of microbiomes from stool samples of multiple human donors. For this study, we capitalized on powerful plant resource populations comprising multiple pairs of NILs in which the waxy trait was bred into different elite parental backgrounds, thus providing biological replication of the waxy trait across multiple genetic backgrounds. Grains from these lines was used for both *in* *vitro* and *in* *vivo* studies to examine the influence of starch composition on the human gut microbiome.

Results from our *in* *vitro* fermentation study revealed significant differences in the effects of waxy versus wild type grain (treatment effects) on ecological metrics (α- and β-diversity), taxonomic features, and metabolite profiles. An unexpected finding from the *in* *vitro* fermentation studies was the consistency of treatment effects on butyrate production and abundances of specific amylolytic, butyrate-producing taxa that were detected across diverse microbiomes of the different human donors. Different genera of the family *Lachnospiraceae* were consistently less abundant in fermentations with amylose-deficient waxy sorghum. This conserved microbiome signature effect was particularly strong for *Roseburia*, which was significantly reduced in fermentations of waxy grain from all 12 of the donor microbiomes tested. This finding is of particular interest because increased abundances of *Roseburia* have been associated with reduced susceptibility to inflammatory and metabolic diseases.^44–46^ *Roseburia* species are known to degrade starch^47^ and produce butyrate as a primary end-product of fermentation.^48^ Moreover, our molecular complementation studies confirmed that the amylose-enriched RS purified from wild type lines alone could complement the defects of waxy grain in promoting growth of *Roseburia* and butyrate production. We also highlight a number of different human feeding studies where dietary supplementation with RS was associated with increases in *Roseburia* in the gut microbiome.^39,49,50^ Thus, despite the differences in ecological environments of *in* *vitro* fermentations and human feeding studies, the *in* *vitro* fermentation model may be relevant for mechanistic studies of *Roseburia* species in humans.

Although our *in* *vitro* fermentation data was quite compelling, we wanted to further determine if a significant treatment effect of the different starches (waxy versus parental) in a whole grain context could also be detected in an animal model in which the same human microbiomes we evaluated *in* *vitro* were present in an intact gastrointestinal environment. We therefore chose to test for these effects in human microbiota-associated (HMA) mice (germ-free mice inoculated with a donor human gut microbiome). Although HMA models have their limitations, including genetic, behavioral, physiological, and anatomical differences from humans,^36,51^ they do however allow for examination of the complex interactions between human microbiotas and dietary components and the potential for dietary modulation of the microbiome to influence host phenotypes. Such studies are not possible with conventional mice harboring a mouse microbiome because of the divergence in species composition between mouse and human microbiomes.^52^ In our studies, feeding diets supplemented with whole-grain sorghum from isogenic parental versus waxy lines revealed significant treatment effects on the gut microbiome. Notably, the mouse diet fabrication process limited the amount of whole grain sorghum that could be introduced to 20% whole grain sorghum from the isogenic pair of wild type/waxy-derivative lines. This amount equates to only a modest difference in amylose content between the two diets of approximately 2% (in the wild type line), whereas studies with purified substrates typically use higher concentrations of RS or other fibers (e.g., 10–30%).^24,53^ Nonetheless, even with this modest difference in dietary amylose content, we detected significant treatment effects of diet (waxy versus wild type) on overall compositional features of the microbiome as well as changes to several individual taxa. Treatment effects were significant across all four HMA mouse lines, but the differences in taxonomic configurations were unique to a given donor microbiome, a result that is often observed in experiments with HMA mice.^38,39^

Perhaps the most intriguing outcomes from the feeding experiments in HMA lines were that (i) some taxonomic differences between waxy and wild type sorghum diets were shared across HMA mouse lines from more than one donor and that (ii) animals fed waxy diets had statistically significant increases in weight gain, regardless of the donor microbiome. With respect to taxonomic effects, genera such as *Muribaculaceae, Anaerostipes, Eubacterium, Agathobacter, Blautia, Erysipelatoclostridium*, and *Phascolarctobacterium* showed significant effects of treatment (diet) in two or more of the HMA mouse lines, but the directionality of the effects for these taxa were not necessarily consistent across lines, suggesting their responsiveness is dependent on context of the microbiome. In contrast, the genus *Faecalibacterium* and members of the taxonomic group *Christensenellaceae R-7* group showed consistent effects in the same direction, with significantly lower in abundances in animals fed amylose-depleted waxy sorghum diets in two or more of the HMA mouse lines. Both organisms are well-regarded as beneficial microbes. For example, abundances of *Christensenellaceae* are associated with longevity,^54^ reduced susceptibility to inflammatory bowel diseases,^55^ and positive effects on body mass index^56^ while the genus *Faecalibacterium* is associated with human health benefits and negatively correlates to inflammatory bowel disease.^57–60^ Studies comparing weight gain from consumption of wild type and waxy grains are limited, but one study in broilers demonstrated significant increases in weight gain in animals fed grain from waxy versus non-waxy hybrids of maize.^61^

The microbial taxa showing significant responses to diet in the HMA mice were largely distinct from the taxa affected *in* *vitro*. This result is not surprising since the *in* *vitro* fermentation conditions are quite different from the mouse gut ecosystem. In particular, *Roseburia* failed to show significant responses to diet in the HMA lines, despite their strong responsiveness in *in* *vitro* fermentations. This outcome may be due to challenges with getting *Roseburia* species from the human microbiomes to efficiently colonize the mouse gut in our HMA model. Attempts to quantify *Roseburia* species in the HMA lines with our species-specific qPCR assays confirm this hypothesis, with most species colonizing at levels < 10^5^ CFU/g of feces and some declining to even lower levels over time (Supplemental Figure 4). In contrast to the situation with *Roseburia*, other families containing prominent amylolytic members such as genera *Faecalibacterium* from the *Ruminococcaceae* family showed consistent and significant responses to the dietary treatments in the HMA mouse lines from multiple microbiomes (Figures 2 and 7). In this instance, it may be that composition of the media for our *in* *vitro* fermentations favors growth of members of the *Lachnospiraceae*. While we absolutely expect there to be differences in responsiveness of organisms under *in* *vitro* conditions versus in vivo in HMA mouse lines, the focus of our study and the more relevant outcome is that both *in* *vitro* and *in* *vivo* experimental approaches showed that grain from wild type and waxy derivatives causes significant differences in the microbiome.

Waxy starch traits have been identified and/or introduced by conventional breeding in many small grain crop species,^6–11^ and these traits also occur naturally in locally cultivated and consumed varieties of rice that are a staple food in some geographies.^16^ In the instances where it has been measured, waxy grains have higher ratings on the glycemic index (i.e., propensity for increasing blood glucose levels) than non-waxy, largely due to the higher levels of readily digestible amylopectin in waxy lines.^16,17^ There could be significant, long-term implications for health outcomes in populations consuming waxy grain on a regular basis. In addition to the potential physiological consequences of diets containing grains that rate high on the glycemic index, our data supports the conclusion of the waxy trait also has undesirable effects on the microbiome in different commodities. The absence or decreased levels of amylose in waxy lines reduces RS content and the beneficial effects of RS on the gut microbiome, thus essentially decreasing a significant fiber benefit of the grain. Recent studies of the glycemic index for a broad panel of rice cultivars, including improved cultivated varieties, showed a strong association of naturally-occurring waxy alleles with elevated glycemic index values and a significant negative correlation between amylose content and glycemic index rating.^16^ Studies have also showed significant associations between amylose content, predicted glycemic index and glycemic load, and allelic variation linked to waxy variants.^29,30^ More detailed studies of health outcomes and microbiome composition in populations consuming large amounts of waxy or high-amylose rice could therefore illuminate the potential for waxy rice consumption to predispose the local populations to type II diabetes.^17,30^ More research needs to be done to determine the negative ramifications of consuming high quantities of waxy grain.

With respect to crop improvement, there is growing recognition that traits such as glycemic index should be included as targets for improvement, and high-throughput methods for phenotyping glycemic index and for microbiome have been reported.^62,63^ Given our results and the known beneficial effects of RS on the human gut microbiome and human health, we further posit that high-throughput methods for microbiome phenotyping, such as *in* *vitro* fermentation, can also be incorporated into improvement programs, permitting improvement of crops for traits that may also be associated with human health.

## Conclusions

The findings of this study revealed that the wide use of waxy starches in food products could potentially have significant undesirable effects on the composition and function of the gut microbiome compared to foods with native, amylose-containing starches. Although our study does not permit direct inference of the effects of waxy starch on human health, the organisms most affected by waxy grain in *in* *vitro* fermentations (e.g., *Roseburia*) and the HMA mouse model (e.g., *Faecalibacterium*) are well regarded as beneficial microbes. These undesirable microbiome effects of waxy grain were also accompanied by a weight gain phenotype in HMA mice. Thus, it is reasonable to hypothesize that long-term consumption of waxy grains may have multiple unintended and undesired effects that are relevant to health. Our work illuminates the need to better understand the potential trade-offs between functional traits and effect on microbiome, and for plant breeders and food scientists to consider these trade-offs when developing hybrids for food ingredients and food product formulations.

## Materials and methods

### Grain information

The information of the grains used in this study are provided in Table 1. Six waxy grain sorghum lines and their near iso-genic wild type lines were obtained from the U.S. Department of Agriculture, Agricultural Research Service (USDA-ARS), Wheat, Sorghum and Forage Research Unit (Lincoln, NE). The amylose content in sorghum were determined using a commercial kit according to the manufacturer’s instructions (K-AMYL Amylose/Amylopectin Assay Kit, Megazyme, Wicklow, Ireland).

### *In vitro* digestion

Whole grain samples were digested following established procedures.^64^ Briefly, 5 g of whole grain were ground into fine powder using Geno/Grinder 2025 (SPEX SamplePrep) at 1600 rpm for 10 min. Then, 2.5 g of sample was mixed with 30 mL of water in a 50 mL Falcon tube for 20 min until fully dispersed. Tubes containing the slurries were immersed in boiling water for 20 min with constant shaking. The slurries were then placed on an orbital shaker (200 rpm) and incubated at 37°C for 40 min. The pH was adjusted to 2.5 with 1 M HCl followed by the addition of 1 mL of 10% (wt/vol) pepsin (P7000; Sigma, St. Louis, MO) in 50 mM HCl. The slurry was then incubated on an orbital shaker (200 rpm) at 37°C for 60 min. The pH was adjusted to 6.9 with 0.5 M NaHCO_3_ and 5 mL of 12.5% (wt/vol) pancreatin (P7545; Sigma, St. Louis, MO) in 0.1 M sodium maleate buffer, and 0.2 mL of amyloglucosidase (E-AMGDF, 3,260 U/mL, Megazyme) was added. The slurry was incubated for 6 h at 37°C with orbital shaking at 200 rpm. Following digestion, the material was transferred to dialysis tubing (molecular weight cutoff of 1000 Dalton) and dialyzed against distilled water for 3 days at 4°C with a water change every 12 h. The retentate from the dialysis was collected, freeze-dried and resuspended in 30 mL of sterile distilled water. The resistant starch concentration was determined with a commercial kit using the protocol variation entitled ‘Determination of total starch content of samples containing resistant starch’ (K-TSTA, Total Starch Assay Kit (AA/AMG), Megazyme).

### Fecal donor and *in* *vitro* fecal fermentation

Fresh fecal samples from 12 healthy adults with no history of gastrointestinal abnormalities and no prebiotic, probiotic, or antibiotic consumption within the past six months were collected using a commode specimen collection kit (Fisher Scientific, NH, USA). All procedures involving human subjects were approved by the Institutional Review Board of the University of Nebraska–Lincoln before initiating the study (20160816311EP). A 1:10 fecal slurry was prepared in an anaerobic chamber (5% H_2_, 5% CO_2_, and 90% N2; Bactron X, Sheldon Manufacturing, Cornelius, OR, USA) within 2 h of collection by adding sterile 10% glycerol in phosphate-buffered saline, pH 7.0 (1:9, w/v) and mixing with a stomacher for 4 min prior to storing at −80°C until fermentation.

*In vitro* batch fermentations were performed inside an anaerobic chamber. Two hundred fifty microliters of resuspended sample was mixed in a 1 mL-deep well in a 96-well plate with 0.25 mL of 2X fermentation medium containing (per liter): 1 g Bacto casitone,1 g yeast extract, 2 g K_2_HPO_4_, 3.2 g NaHCO_3_, 3.5 g NaCl, 1 mL hemin solution (KOH 0.28 g, 95% ethanol 25 mL, hemin 100 mg and ddH_2_O to 100 mL), 0.05 g bile salts, 0.5 g/L cysteine HCl, 0.6 mL resazurin (0.1%), 10 mL ATCC trace mineral supplement, 3.6 mL VFA solution (17 mL acetic acid, 1 mL n-valeric acid, 1 mL iso-valeric acid, 1 mL iso-butyric acid mixed with 20 mL of 10 mM NaOH), 10 mL ATCC vitamin supplement and 1 mL vitamin K-3 solution (0.14 g vitamin K-3 in 100 mL 95% ethanol).^65^ The substrate was then reduced at 4°C in the anaerobic box with anerobic gas generator (Mitsubishi™ AnaeroPack-Anaero, Japan) for 3 days before inoculation with 0.05 mL of fecal slurry. *In vitro* fermentations were incubated at 37°C for 16 h.^66,67^ After fermentation, samples were centrifuged at 4000 *g* for 10 min. Pellets and supernatants were stored at −80°C until further processing.

### Starch complementation experiment

Starch from wild type lines ‘Wheatland’ and ‘Tx430’ was extracted using a modified protocol from Xie et al.^68^ The isolated starch then underwent *in* *vitro* digestion as described previously.^64^ The resulting starch concentration was determined as described above. The digested starch was added to digested waxy sorghum substrate (starch concentration: waxy Wheatland, 2.79 mg/100 mL; waxy Tx430, 2.24 mg/100 mL) to adjust the starch concentration to that of the corresponding wild type sorghum substrate (starch concentration: wild type Wheatland, 12.82 mg/100 mL; wild type Tx430, 9.85 mg/100 mL), thus making the starch concentration 9.31 mg/100 mL for supplemented waxy Wheatland and 6.57 mg/100 mL for supplemented waxy Tx430. Waxy sorghum, waxy sorghum supplemented with starch and wild type sorghum were each inoculated into one of six human fecal microbiomes with the largest and smallest differences between waxy and wild type sorghum observed during *in* *vitro* fermentations (largest and smallest R^2^ value from PERMANOVA analysis, data not shown) as described above.

### Human microbiota-associated mice

Female germ-free C57BL/6 mice were born and reared in flexible film isolators and maintained under gnotobiotic conditions (temperature 20°C, relative humidity 60%, 14 h light/10 h dark cycle) at the University of Nebraska-Lincoln. Germ-free status of the breeding colony was checked routinely as described.^24^ The Institutional Animal Care and Use Committee at the University of Nebraska-Lincoln approved all procedures involving animals (protocol 1700). At 12–13 weeks of age, germ-free mice were transferred from isolators to autoclaved individually ventilated cages mounted on racks with positive airflow as previously described^24^ and then colonized immediately with human stool microbiomes. Four human fecal microbiomes exhibiting the greatest differences between waxy and wild type sorghum during *in* *vitro* fermentations (largest R^2^ value and smallest P value from PERMANOVA analysis, data not shown) were selected for inoculation into mice. To establish human microbiome-associated (HMA) mice, 100 uL of each human fecal slurry (*n* = 18 mice for each donor) was orally gavaged into mice once. Mice were then randomly assigned to dietary treatments based on body weight at the time of colonization and housed three per cage by donor microbiome. No significant differences in body weight were observed among treatment groups on the day of randomization (data not shown). Fecal pellets were collected from individual mice twice a week and stored at −80°C until DNA extraction. Cecal contents were collected at necropsy and stored at −80°C until DNA extractions were performed as previously described.^69^ Serum was also collected at necropsy and later analyzed using a Milliplex Mouse Cytokine/Chemokine Magnetic Bead Premixed 32-Plex Kit (Milliplex; Millipore, Billerica, MA) and a MAGPIX instrument (Luminex Corporation, Austin, TX) as per the manufacturer’s instructions.

### Experimental diets

Mice were fed an autoclaved chow diet (LabDiet 5K67, Purina Foods, St. Louis, MO) ad libitum prior to the introduction of experimental diets at the time of colonization with human stool microbiomes. Experimental diets were prepared and irradiated by Research Diets, Inc. (New Brunswick, NJ). Mice were fed either a low fiber control diet based on the AIN-93 G rodent diet formulation with dextrose as the sole carbohydrate source or a customized diet based on AIN-93G where the dextrose was replaced with 20% Wheatland sorghum flour from either wild type or waxy isogenic line. Six mice for each donor microbiome and diet combination were fed for four weeks. Diet compositions are listed in Supplementary Table 2.

### DNA extraction and 16S rRNA gene sequencing

DNA was extracted from mouse fecal pellets and cecal contents using the BioSprint 96 workstation (Qiagen, Germantown, MD) and the BioSprint 96 one-for-all Vet kit with the addition of buffer ASL (Qiagen) and bead beating.^63^ The V4 region of the bacterial 16S rRNA gene was amplified from each sample using the dual-indexing sequencing strategy.^70^

### 16S rRNA gene sequencing processing

Paired-end sequences were analyzed using Quantitative Insights Into Microbial Ecology (QIIME) program (version 2).^71^ Sequences were truncated (220 bases for forward reads and 160 bases for reverse reads) and denoised into amplicon sequence variants (ASVs) using DADA2.^72^ All ASVs were assigned with taxonomic information using pre-fitted sklearn-based taxonomy classifier SILVA database (release 138)^73^ and were then binned at genus level and transformed to relative abundance by dividing each value in a sample by the total reads in that sample. PICRUSt2 was used to generate predicted metagenomes.^74^

### Investigation of *Roseburia*

To quantify individual *Roseburia* species responses to waxy sorghum, we used qPCR to identify four *Roseburia* species. Representative genomes of type strains *Roseburia intestinalis* DSM 14610, *Roseburia inulinivorans* DSM 16841, *Roseburia hominis* DSM 16839 and *Roseburia faecis* DSM 16840 were downloaded from the NCBI database (http://www.ncbi.nlm.nih.gov/genome) and analyzed using dbCAN2^75^ to investigate the functions of carbohydrate utilization. Species-specific primers were designed using Rapid identification of PCR primers for unique core sequences (RUCS).^76^ Primer specificity was validated by Primer BLAST against the NCBI RefSeq representative genome database.^77^ The validated species-specific primer sequences used were: *Roseburia intestinalis* F-CGAAGCACTTTATTTGATTTCTTCGG, R- TTTTTCACACCAGGTCATGCG; *Roseburia hominis* F- AAGTCTTGACATCCCACTGACA, R- CACCACTGCTCCGAAGAGAA; *Roseburia inulinivorans* F- GACATCCTTCTGACCGGACAG, R- GGCTACTGGGGATAAGGGTTG; *Roseburia faecis* F- CGCAACCCCTGTCCTTAGTAG, R- AGATTTGCTCGGCCTCACG. All primers were synthesized by Integrated DNA Technologies (Coralville, IA). Real-time PCR reactions were prepared in a 10 µL volume containing 5 µL 2x SYBR Green, 3 µL nuclease free water, 1 µL primer mix (a mixture of forward and reverse primer of 5 µM each), and 1 µL DNA template. Thermocycling conditions included (i) an initial denaturation step of 5 min at 95C; (ii) 40 cycles of 20 s at 95^◦^C, 25 s at annealing temperature (61^◦^C for *R. faecis* and *R. inulinivorans* specific primers; 63^◦^C for *R. intestinalis* and *R. homonis* specific primers), and 30 s at 72^◦^C; (iii) one cycle of 15 s at 95^◦^C; (iv) one cycle of 30 s at 60^◦^C; and (v) one 20-min interval to generate a melting curve. The cycle threshold of each sample was then compared to a standard curve made by diluting genomic DNA from type strain *Roseburia intestinalis* DSM 14610, *Roseburia inulinivorans* DSM 16841, *Roseburia hominis* DSM 16839, and *Roseburia faecis* DSM 16840.

### SCFA analyses

SCFA (acetate, propionate, and butyrate) and branched chain fatty acids (BCFA; iso-butyrate and iso-valerate) from fermentation samples were analyzed by gas chromatography as described previously.^69^

### Statistical analysis

All analyses were performed using R and RStudio.^78,79^ Bacterial community diversity was assessed by α-diversity and β-diversity. α-diversity metrics, including Shannon index, bacterial richness (total ASV numbers) and Pielou’s Evenness and β-diversity in Bray–Curtis distance, were calculated using rarefied ASV data with the phyloseq and vegan packages.^80,81^ Differences in α-diversity were compared by paired Wilcoxon tests. Differences in the microbiome communities were compared by PERMANOVA using the Adonis function in vegan. In the complementation study, differences in Bray–Curtis distance between different groups to waxy sorghum groups were compared by Kruskal–Wallis test followed by post hoc pairwise multiple comparisons using Dunn’s test. Differential abundance analysis was also performed using LEfSe (linear discriminant analysis effect size).^82^ Bacterial genera and *Roseburia* species were compared by paired-Wilcoxon test for two groups or Kruskal–Wallis test followed by Dunn’s test for more than two groups.

Differences in *in* *vitro* SCFA production and weight gain in mice were compared by repeated measures ANOVA with FDR corrections. Spearman’s correlation analysis was performed between fecal SCFA concentrations and the abundances of genera, and between key microbiome features (abundances of genera and *Roseburia* species and SCFA concentrations) and starch concentration in the complementation experiment. Pearson’s correlation analysis was performed between the difference in average weight gain between mice fed with waxy versus wild type sorghum and average relative abundance difference of bacterial genera. Data was visualized using different programs and R packages.^83–86^

## Supplementary Material

Supplemental Material

## Data Availability

The sequence data reported in this paper have been deposited in the NCBI database (PRJNA811321).
