# Supplementary figures and images for: The waxy mutation in sorghum and other cereal grains reshapes the gut microbiome by reducing levels of multiple beneficial species
Source: Gut Microbes. 2023 Feb 21;15(1):2178799. doi: 10.1080/19490976.2023.2178799 (PMC9980621; doi:10.1080/19490976.2023.2178799)

Weighted UniFrac Distance

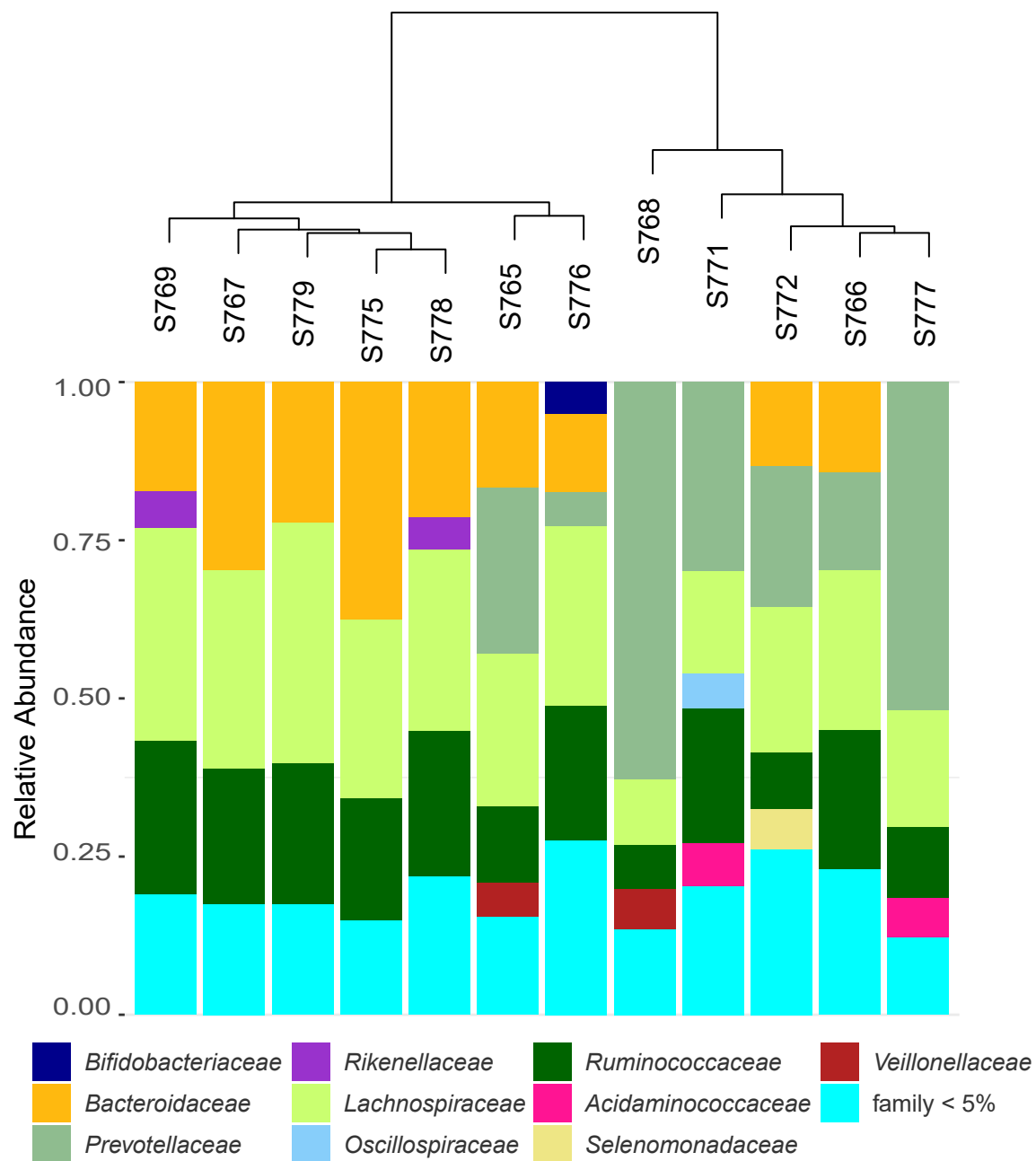

Supplement: Supplemental Material [file KGMI_A_2178799_SM2226.zip › Supplemental Figure 1.pdf]

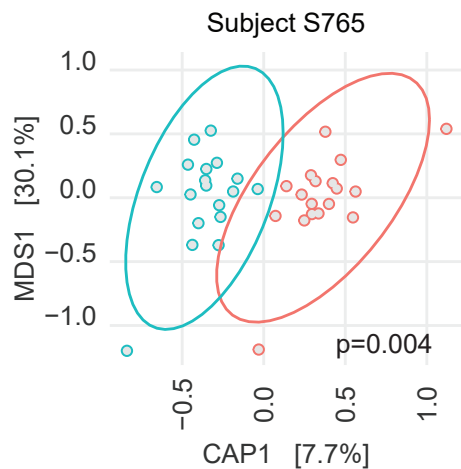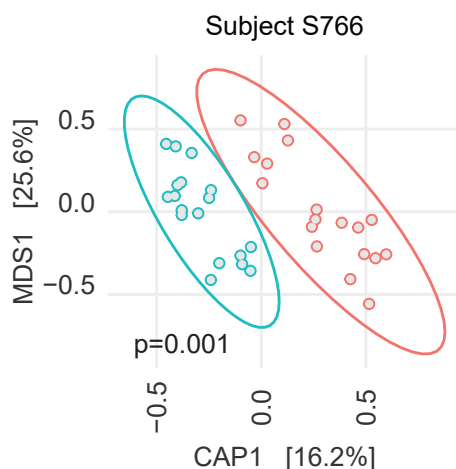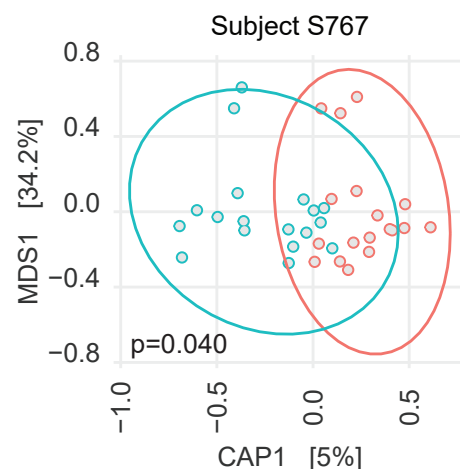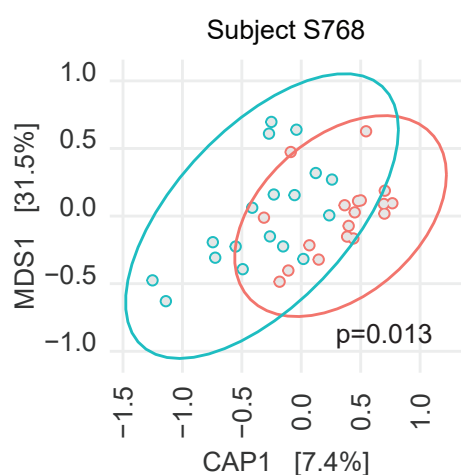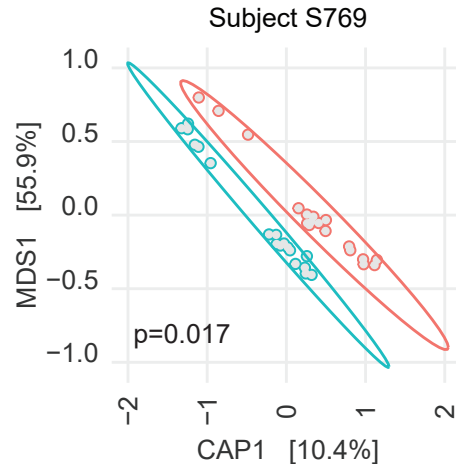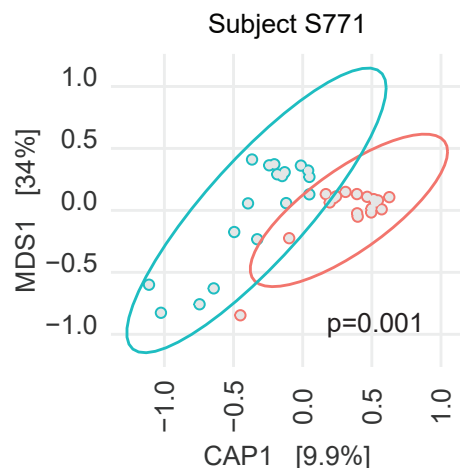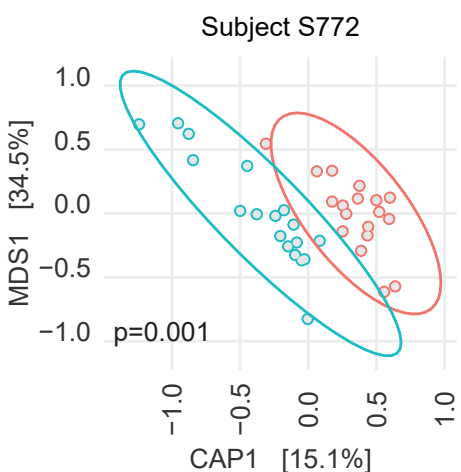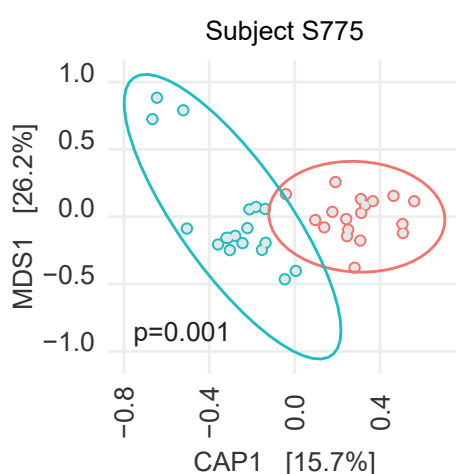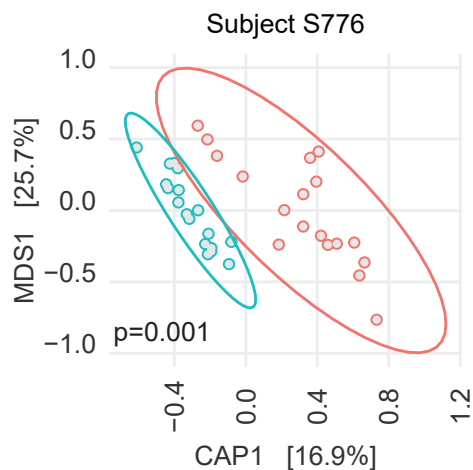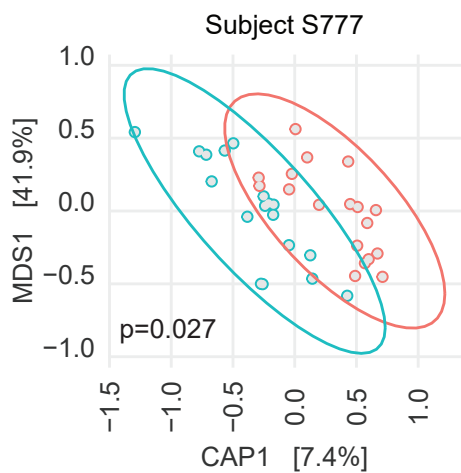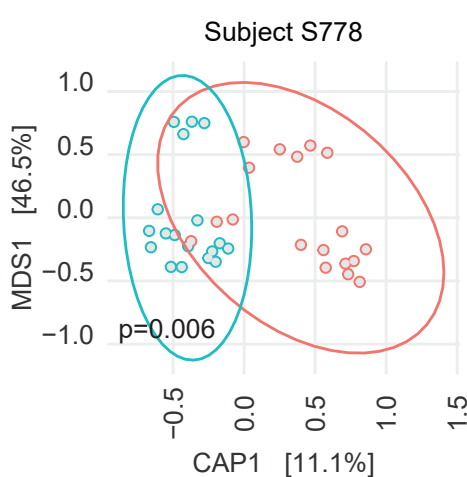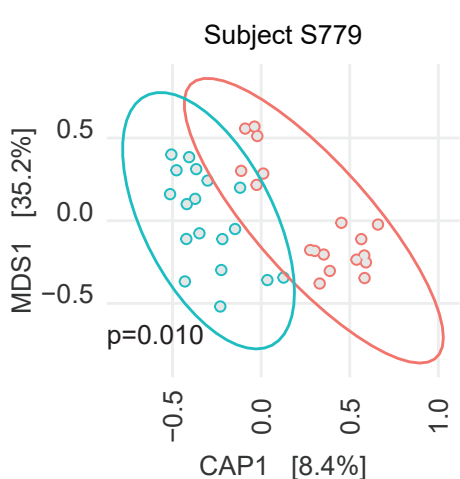

Supplement: Supplemental Material [file KGMI_A_2178799_SM2226.zip › Supplemental Figure 2.pdf]

Subject S765

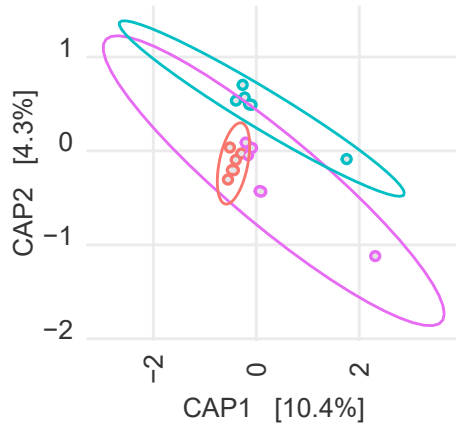

Subject S766

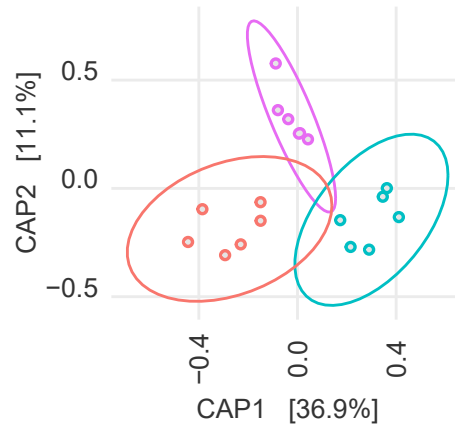

Subject S767

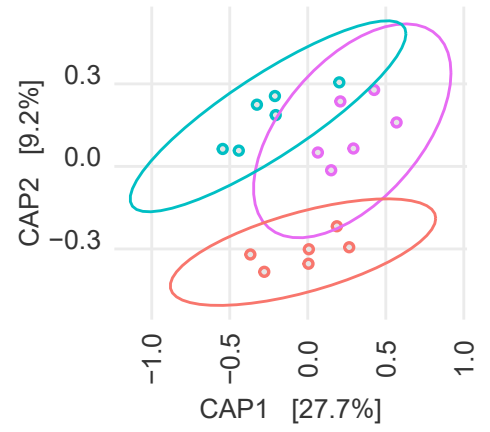

Subject S776

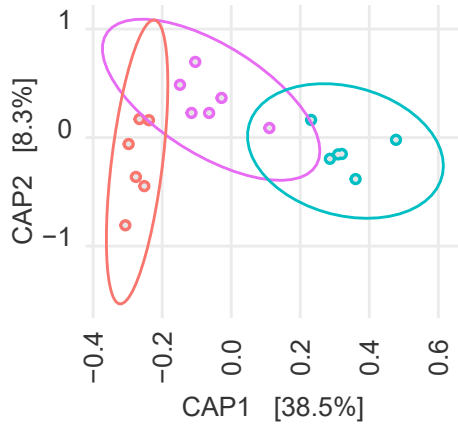

Subject S778

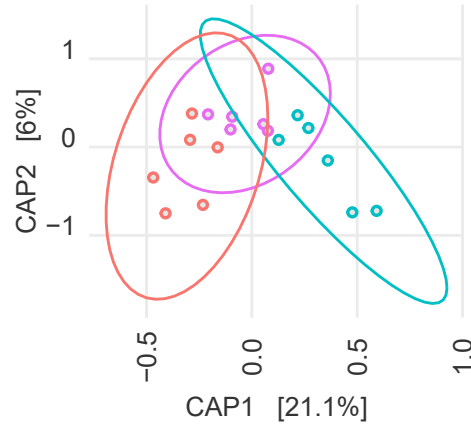

Subject S779

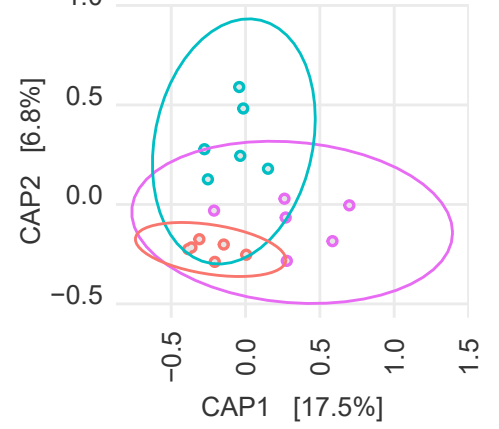

— Wild type  
— Waxy  
— Waxy+Starch

Supplement: Supplemental Material [file KGMI_A_2178799_SM2226.zip › Supplemental Figure 3.pdf]

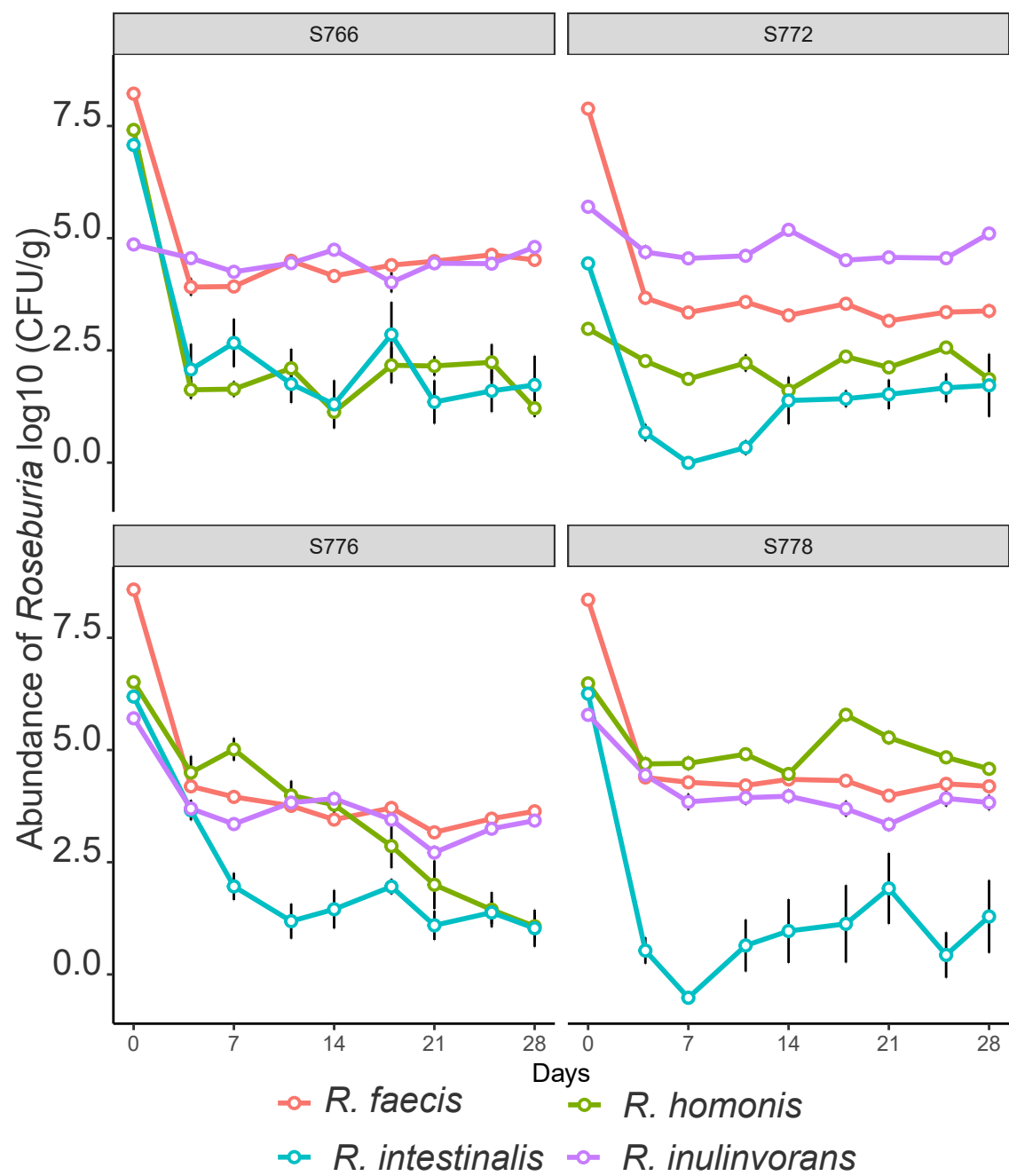

Supplement: Supplemental Material [file KGMI_A_2178799_SM2226.zip › Supplemental Figure 4.pdf]

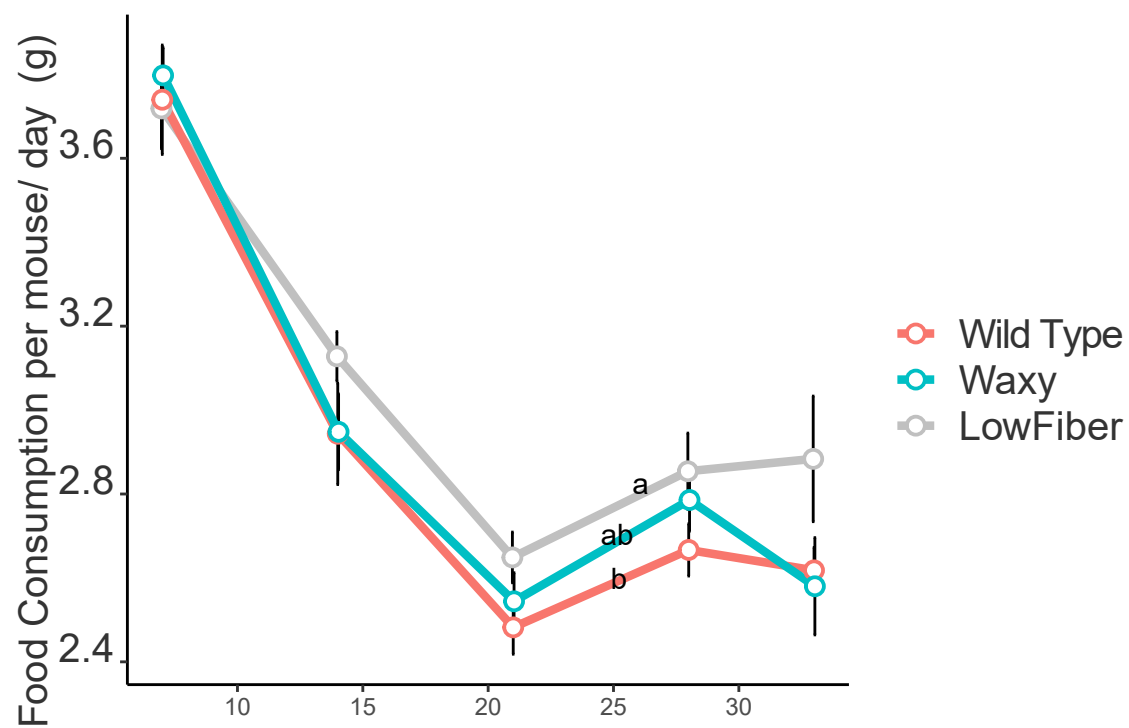

Supplement: Supplemental Material [file KGMI_A_2178799_SM2226.zip › Supplemental Figure 5.pdf]

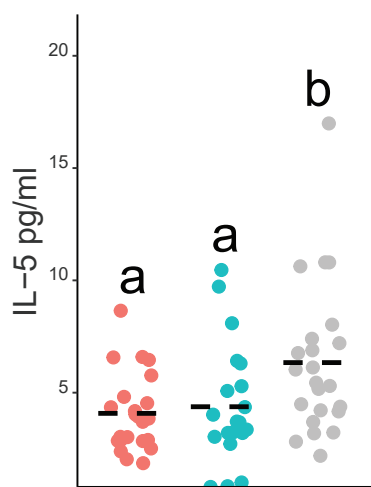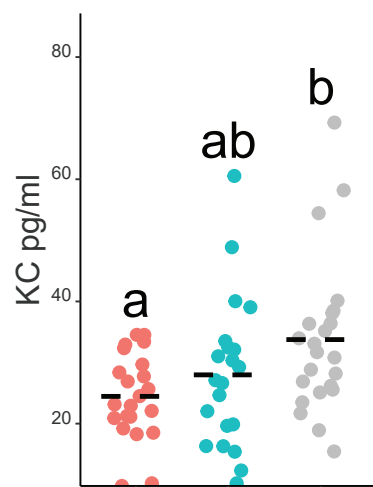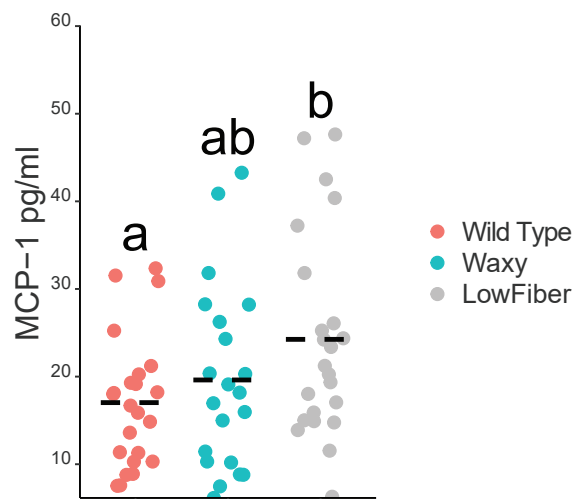

Supplement: Supplemental Material [file KGMI_A_2178799_SM2226.zip › Supplemental Figure 6.pdf]
